# Malignant myoepithelioma of the external auditory canal — a rare case report with literature review and clinical importance of foramen of Huschke

**DOI:** 10.1186/s12957-024-03317-5

**Published:** 2024-01-24

**Authors:** Zuzana Mateášiková, Richard Salzman, Jaroslav Michálek

**Affiliations:** 1 Department of Otorhinolaryngology and Head and Neck Surgery, Faculty of Medicine and Dentistry, Olomouc University Hospital, Palacký University Olomouc, Olomouc, Czech Republic; 2 Department of Pathology, Faculty of Medicine and Dentistry, Olomouc University Hospital, Palacký University Olomouc, Olomouc, Czech Republic

**Keywords:** Myoepithelial tumor, External ear canal, Ear cancer, Infratemporal fossa

## Abstract

**Background:**

A malignant myoepithelioma is a rare tumor, mostly arising from the salivary glands. Myoepitheliomas of the ear have rarely been reported. The manuscript reports myoepithelial carcinoma of the external auditory canal (EAC) spreading to the infratemporal fossa. A clinician must be aware of anatomical variation of the bony EAC wall, such as the foramen of Huschke. This rare defect may be a pathway for spreading pathologies between these two anatomical regions.

**Case report:**

We present a case of osteoma-like stenosis of the EAC, which turned out to be an extremely rare malignant tumor. The preoperative MRI and PET/CT revealed that two parts of the tumor communicated through a defect in the antero-inferior portion of the bony ear canal. No distant metastases were detected. Subsequently, the tumor was resected from the ear canal and the infratemporal fossa en bloc. Perioperatively the defect in the EAC wall was suspected of the foramen of Huschke. After the surgery, the older scans of the patient from the past showed no presence of a congenital EAC wall defect. Therefore, the authors concluded that the tumor aggressively grew through the bone due to its biological nature.

**Conclusion:**

Malignant myoepithelioma of the external auditory canal is an extremely rare condition and could be misdiagnosed as other benign lesions. In cases of suspicious lesions, it is advisable to do a probatory biopsy from the EAC. Surgery is the treatment of choice in malignant myoepitheliomas, and regular follow-ups are essential to monitor for recurrence or metastatic disease. Any mass located at the antero-inferior portion of the EAC wall warrants close evaluation due to its potential for expansion from the EAC.

## Background

Myoepithelial tumors are rare neoplasms, both benign and malignant. They mostly arise from salivary glands. However, they also occur in the skin, breast, sinonasal region, or larynx. A few cases involving the infratemporal fossa and orbit have been described [1–3]. The first myoepithelioma of the external ear canal was described by Chen in 1982 [4]. Malignant myoepithelioma of the ear has rarely been reported [5–7].

The EAC could have a possible communication with the infratemporal fossa through the foramen of Huschke. The foramen of Huschke is a developmental defect located at the antero-inferior portion of the osseous EAC wall and posteromedial part of the temporomandibular joint (TMJ) area [8, 9]. We reflect on the clinical importance of this unusual communication by highlighting a rare pathology of the EAC with the proposal for its diagnostic management.

## Case presentation

**Fig. 1:**
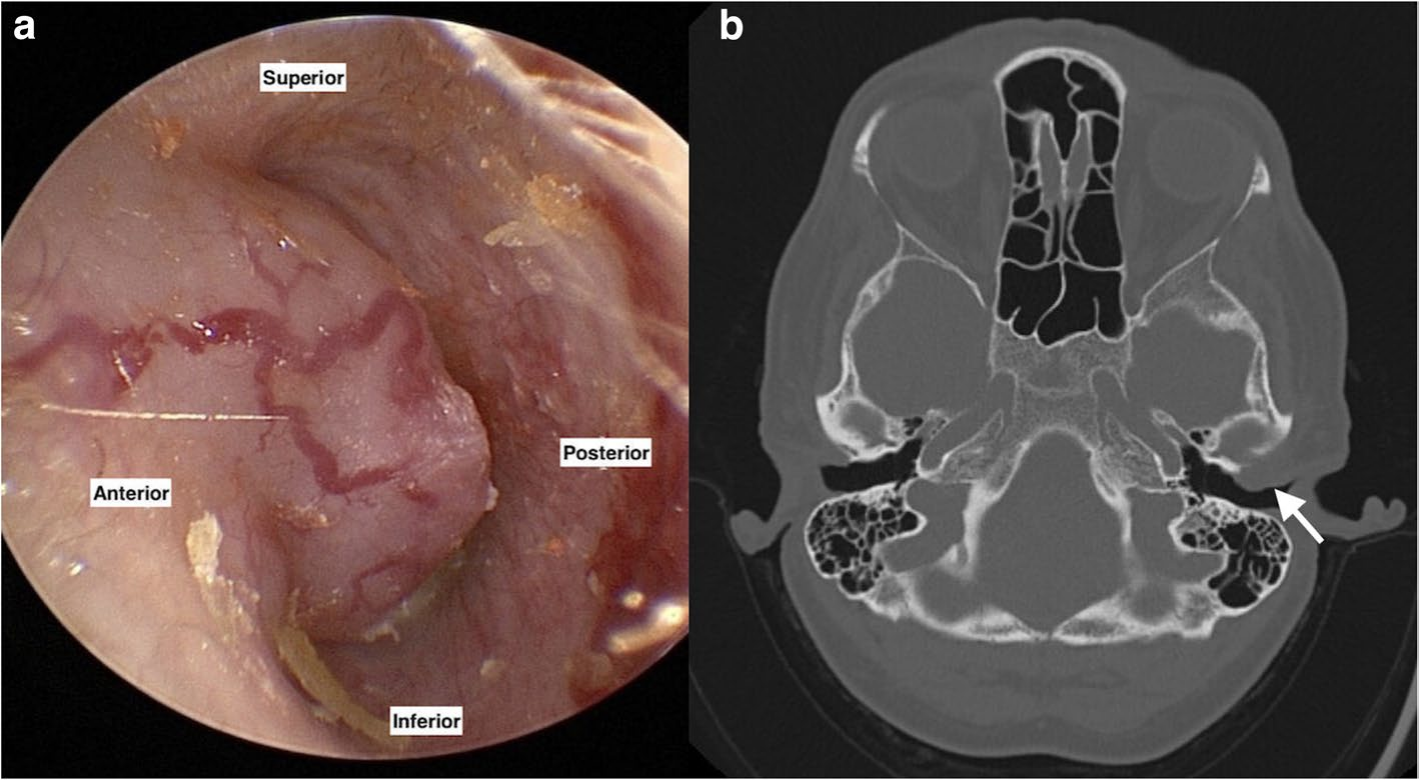
**A** The endoscopic view of the left ear canal with myoepithelial tumor, anatomical orientation marked (left image). **B** The preoperative CT scan showing the pathological formation (20 × 11 mm) in the anterior EAC wall (arrow), with the marginal osteolytic process (right image)

**Fig. 2:**
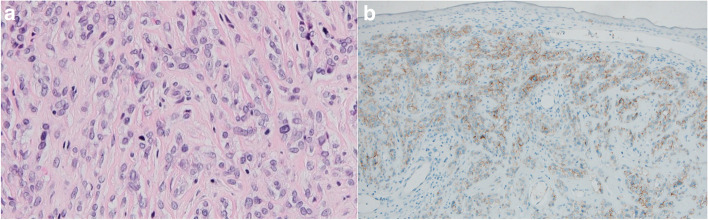
**A** Myoepithelial carcinoma in hematoxylin–eosin stain, 200 × magnification (left image). **B** Myoepithelial carcinoma in BerEP4 immunohistochemistry staining, 100 × magnification (right image)

We present a case of a 54-year-old woman who attended the outpatient clinic of the tertiary referral center, in March 2021, with a two-month history of blunt ear pain, aural fullness, and muffled hearing in her left ear. Through clinical examination, a solid mass at the anterior ear canal wall was revealed imitating an osteoma at first sight but with highly vascularized overlying skin (Fig. 1A). There was no palpable resistance in the neck, parotid, or mastoid regions. The patient’s pure tone audiogram was normal. Because of the atypical appearance and location of the lesion, a probatory biopsy taken from the ear canal mass revealed malignant myoepithelioma with myoepithelial differentiation (Fig. 2A). No ductal differentiation was detected. Immunohistochemistry showed positivity for cytokeratin, smooth muscle antigen, calponin, S-100 protein, and a BER-EP4 (Fig. 2B).

CT scan showed the nonhomogeneous formation measuring 20 × 11 mm in the anterior EAC wall, with the marginal osteolytic process. This formation was located dorsally to the TMJ (Fig. 1B).

**Fig. 3:**
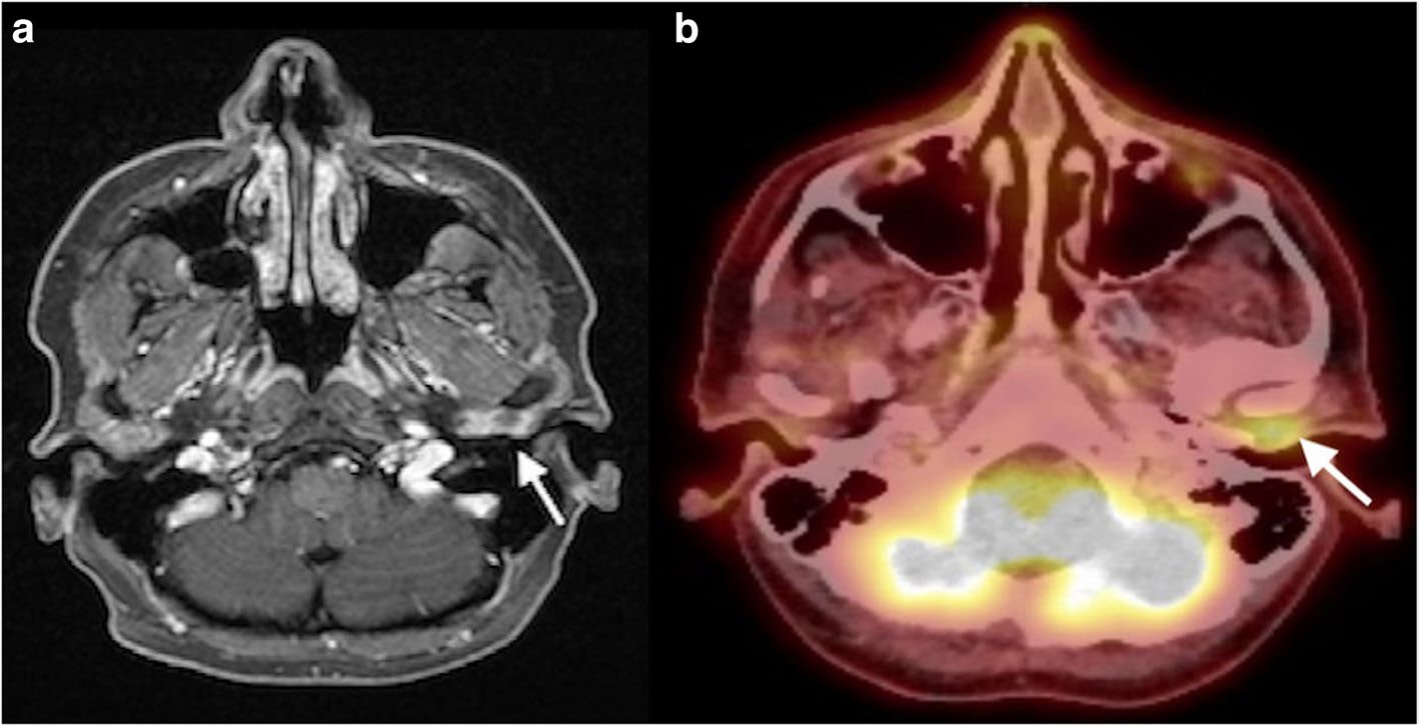
**A** The preoperative T2 weighted axial magnetic resonance image showing a pathological enhancement (13 × 8 × 10 mm) between the anterior ear canal wall and temporomandibular joint (arrow), (left image). **B** The preoperative PET/CT axial scan showing a small FDG enhanced area (arrow) in the anterior wall of the left external auditory canal (right image)

Magnetic resonance imaging in T2 weighted sequence (Fig. 3A) showed a signal-enhanced area with central hypodensity in the anterior ear canal wall adjacent to the left temporomandibular joint capsule. The tumor did not infiltrate the TMJ capsule. PET/CT scan (Fig. 3B) confirmed a slightly enhanced area in the anterior ear canal wall (SUV 5). No distant metastases were detected.

Surgery was performed by a team consisting of head and neck and maxillofacial surgeons. The tumor was resected using a combined transmeatal and transparotid approach. Initially, using the transmeatal approach, the tumor distance from the tympanic membrane was easily visualized using a 30-degree 3-mm endoscope. The tumor ear canal portion was resected along with the surrounding ear canal bone using a Rosen knife and a Skeeter drill. During the resection, it was found that there was an osseous defect at the antero-inferior part of the EAC wall. The defect location was suspected of the Foramen of Huschke. Subsequently, using a transparotid approach, the tumor infratemporal portion was identified. The initially planned facial nerve identification was not necessary, due to sufficient distance between the tumor and the nerve trunk. Despite the tumor intimate relation to the TMJ, the TMJ joint capsule was not involved and therefore was left intact. The EAC bony defect was reconstructed with tragal cartilage to prevent joint herniation into the auditory canal.

The postoperative course was completely uneventful. The patient did not complain of any hearing loss or mastication pain but suffered from trismus for 3 weeks.

According to the multidisciplinary tumor board (MDT) recommendation, the patient was not referred for adjuvant therapy and was scheduled for a regular oncological follow-up with repeat scans. Six months post-surgery, October 2021, a minor bulging near the cartilage used for canal defect reconstruction was noted at the anterior wall of the ear canal. The patient remained completely asymptomatic. The subsequent CT scan did not rule out the recurrence of the tumor. An adequate biopsy was taken under general anesthesia. The definitive histology revealed only fibrotic tissue with no signs of malignancy. The MDT board concluded that further resection would not obtain a better surgical specimen, and only follow-up with the scheduled scans was recommended. The last PET/CT scan from March 2023 showed no signs of recurrence. The patient has been followed up every 3 months with a planned PET/CT scan 12 months down the line.

## Discussion

Malignant neoplasms of the external auditory canal (EAC) include squamous cell carcinoma (SCC), basal cell carcinoma, adenoid cystic carcinoma, and ceruminous adenocarcinoma. While primary ear cancer is uncommon, these structures are more frequently affected by cutaneous squamous cell carcinoma (cSCC) from the pinna or metastatic cSCC of the parotid gland [10]. Rare tumors such as myoepitheliomas, benign or malignant, may also affect the ear, including the external meatus [4–7].

So far, only a few cases of myoepithelioma of the ear including the external ear canal have been reported. Firstly, a benign myoepithelioma in the external auditory canal was described by Chen in 1982 [4]. Joseph et al. reported a case of malignant myoepithelioma in the mastoid region arising from the postauricular soft tissue [6]. Later, the malignant myoepithelioma in the external ear canal was described also by Didier et al. [7]. Middle ear involvement by benign large myoepithelioma was described by Hagisawa et al. [11]. The largest malignant myoepithelioma of the ear involving the middle ear, mastoid, and the parotid gland with extension to the infratemporal fossa was described by Saha et al. [5]. According to our best knowledge, this case is the fourth case in the literature describing the malignant myoepithelioma of the ear to date.

Myoepithelial carcinoma is a rare entity composed mostly of neoplastic cells showing myoepithelial but not ductal differentiation. The malignant potential of this carcinoma is intermediate to high-grade. It can arise de novo or from a preexisting tumor, most likely from pleomorphic adenoma [12].

Histologically, myoepithelioma consists almost exclusively of myoepithelial cells [13]. Immunohistochemically they are positive for epithelial markers, e.g., cytokeratins, smooth muscle antigen, calponin, epithelial membrane antigen, and S-100 protein [14]. In our case histopathological findings were consistent with all mentioned above, and a BER-EP4 (antibody against epithelial cell adhesion molecule) was detected, which suggests the cutaneous origin of the tumor [13, 14].

Surgery is the treatment of choice for malignant myoepithelioma. The extension of the surgery is given by tumor origin, size, and affected area based on preoperative radiological findings. The most important indicator of EAC tumor resectability is its growth to the surrounding regions such as the infratemporal fossa, temporomandibular joint, or middle ear.

Malignant myoepitheliomas have a significant local recurrence rate of 42% and a lower risk of distant metastases of approximately 32–40% [3, 15]. However, the final pathology report could also describe infiltrative growth patterns demonstrating the aggressiveness of the tumor. It is likely that malignant myoepitheliomas of the soft tissue are more aggressive and have a higher metastatic potential [14]. Recurrence and metastasis are more common in children even with a negative excision margin [3].

The foramen of Huschke also called tympanic foramen represents a developmental defect of the antero-inferior portion of the osseous EAC wall and posteromedial part of the TMJ area. This rare defect may be a pathway for spreading pathologies between these two anatomical regions [8, 9]. The foramen was named by German anatomist, Emil Huschke, who first described it in 1844. However, the first medical observation was published by the French physician Jean Riolan the Younger almost two hundred years earlier in the seventeenth century [16].

**Fig. 4:**
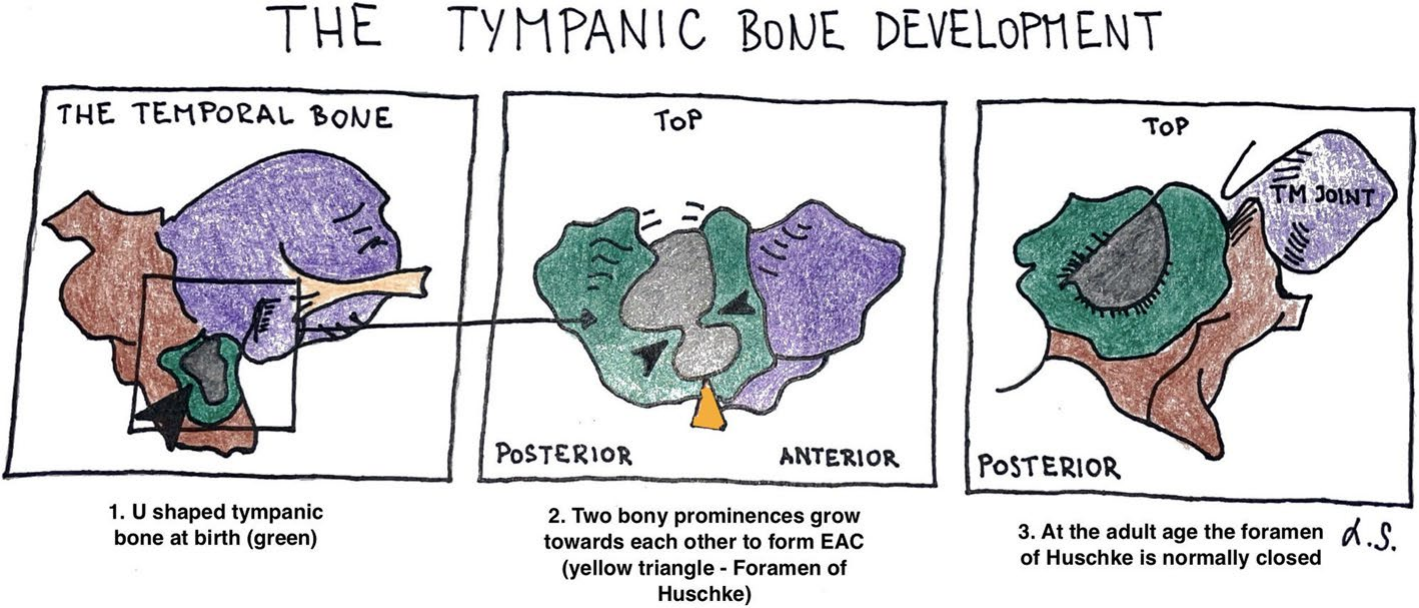
The author’s illustration of the tympanic bone development adjusted and modified (used with permission from Anson BJ, Donaldson JA. Surgical Anatomy of the Temporal Bone*.* 3rd ed. Philadelphia, W.B. Saunders; 1981. p. 122.)

Generally, there is a layer of lamellar bone separating the EAC wall and the TMJ. The lamellar partition of the tympanic bone does not fully close during intrauterine development rather the ossification is completed postpartum. Typically, the anterior and posterior rings of the tympanic bone fuse together by the age of 5 [17]. When the ossification process fails, the missing bone forms the foramen of Huschke (Fig. 4).

The foramen of Huschke is present in up to 20% of the population and can be identified in about 65% unilaterally and in 35% bilaterally [18]. The shape of the tympanic foramen is mostly oval [19]. The mean axial diameter is described as between 3.2 and 4.5 mm, and the sagittal diameter is between 2.4 and 3.6 mm [8]. The definitive presence of the foramen of Huschke is confirmed by a high-resolution CT scan or cone beam CT (CBCT).

The symptoms of the tympanic foramen are nonspecific, and the most common include TMJ herniation to the external auditory canal during mastication, or otologic symptoms like otalgia, clicking tinnitus, aural fullness, or otorrhea, which could indicate an ear canal salivary fistula [17–19].

Nevertheless, infections or tumors can also spread through this bony defect. The classic well-known pathway of spreading out of the EAC is along the fissures of Santorini, which is the most common way of spreading necrotizing external otitis. Santorini fissures are osteocartilaginous or cartilaginous junctions of the EAC [20].

In our case, the tumor originated in the external auditory canal skin and based on the surgical findings, most likely spread through the tympanic foramen into the infratemporal fossa.

After surgery, we searched for previous CT scans to confirm the presence of the foramen since childhood. Unfortunately, the CT scans showed no evidence of a pre-existing tympanic foramen in the osseous part of the ear canal wall. Therefore, the communication between the EAC and the infratemporal fossa was most probably caused by the aggressive growth of the myoepithelial tumor. Furthermore, ear canal tumors, such as SCC, showing infiltrative growth patterns frequently erode the cartilaginous and bony ear canal [10]. This rare localization and behavior of malignant myoepithelioma as a solid mass in the antero–inferior portion of the osseous EAC wall suggests a microscopic foramen or *locus minoris resistentiae* in the bone as the easiest way for the tumor to spread to the TMJ area.

## Conclusion

Malignant myoepithelioma of the external ear canal is an extremely rare condition and could be misdiagnosed for other benign lesions. Preoperative diagnosis may be difficult due to nonspecific clinical manifestation and imaging characteristics, however, a probatory biopsy from the EAC could be reached easily. An appropriate therapeutic approach to the tumor should be based on preoperative radiological findings. Surgery is the treatment of choice for malignant myoepithelioma. Radical tumor excision with preservation of the quality of life remains the main goal of surgery. Regular follow-up after surgery is crucial for patients with malignant myoepithelioma for possible recurrence or metastatic disease. Based on this revealed case, any mass located at the antero-inferior portion of the external ear canal wall should be closely evaluated, in case of its potential expansion from the EAC through a possible existing developmental defect.

## Data Availability

No datasets were generated or analysed during the current study.

## Abbreviations

EAC: External auditory canal
TMJ: Temporomandibular joint
MRI: Magnetic resonance imaging
PET/CT: Positron emission tomography-computed tomography
SUV: Standardized uptake value
MDT: Multidisciplinary tumor board
CT: Computed tomography
SCC: Squamous cell carcinoma
cSCC: Cutaneous squamous cell carcinoma
BER-EP4: Antibody against epithelial cell adhesion molecule
CBCT: Come beam computed tomography
